# Clinical significance of novel biomarkers to predict the natural course of hepatitis B infection

**DOI:** 10.3389/fpubh.2022.1037508

**Published:** 2022-10-28

**Authors:** Weikang Wu, Xiaojie Yuan, Weilu Zhang, Haowei Zhou, Xiangyu Kong, Zhen He, Ting Fu, Wenhua Zhang, Wenling Jia, Chunhui Liang, Haitao Tang, Fengmei Wang, Yancheng Ye, Zhongjun Shao, Zhaohua Ji

**Affiliations:** ^1^Ministry of Education Key Lab of Hazard Assessment and Control in Special Operational Environment, Department of Epidemiology, School of Public Health, Air Force Medical University, Xi'an, China; ^2^Gansu University of Chinese Medicine, Lanzhou, China; ^3^Hepatobiliary Center, Wuwei Cancer Hospital of Gansu Province, Wuwei, China; ^4^Clinical Drug Experiment Institution, Wuwei Cancer Hospital of Gansu Province, Wuwei, China

**Keywords:** hepatitis B, novel biomarkers, phase, HBV RNA, HBcrAg, HBV DNA

## Abstract

**Background and aim:**

Chronic hepatitis B (CHB) can be divided into immune tolerance (IT), immune clearance (IC), hepatitis B e antigen (HBeAg)-negative inactive/quiescent carrier (ENQ), and HBeAg-negative hepatitis (ENH) phases. The conventional biomarkers used to distinguish these phases have limitations. We examined the clinical significance of hepatitis B virus (HBV) RNA and hepatitis B core-related antigen (HBcrAg) as novel biomarkers.

**Methods:**

One hundred eighty-nine patients without treatment currently were categorized by CHB phase (IT = 46, IC = 45, ENQ = 49, ENH = 49). The associations of HBV RNA and HBcrAg with HBV DNA and alanine transaminase (ALT) were analyzed. The decision tree model was used to distinguish the four phases in the natural course of CHB.

**Results:**

The concentrations of HBV RNA and HBcrAg were highest in the IT and IC phases (*P* < 0.01). Serum HBV RNA was similar to HBcrAg in treatment-naïve patients. HBV RNA and HBcrAg correlated with HBV DNA in the HBeAg^+^ and HBeAg^−^ status (HBV RNA: e^+^ r = 0.51, e^−^ r = 0.62; HBcrAg: e^+^ r = 0.51, e^−^ r = 0.71), but their association with HBV DNA differed among phases. The accuracy, sensitivity, and specificity of HBcrAg with ALT in distinguishing the CHB phases were 95.65%, 95.83%, and 95.55%, respectively.

**Conclusion:**

Serum HBV RNA and HBcrAg may be useful to monitor CHB progression.

## Introduction

Chronic hepatitis B (CHB) is such a global health burden that influenced 296 million people worldwide in 2019, with 1.5 million new infections annually (1). Accurate identification of the phases of CHB is important for providing prognostic advice, monitoring disease activity, and determining treatment requirements (2). According to guidelines CHB can be classified into four phases: immune tolerance (IT), immune clearance (IC), hepatitis B e antigen (HBeAg)-negative inactive/quiescent carrier (ENQ), and HBeAg-negative hepatitis (ENH) was based on (hepatitis B surface antigen [HBsAg] and HBeAg), HBV DNA, biochemical tests (alanine transaminase [ALT], and liver biopsy) (3–5).

However, these methods are limited in their ability to distinguish between the phases of CHB. Firstly, to increase the distinction accuracy, serum HBV DNA and ALT are repeatedly monitored; however, this may result in patients not being treated in time (6, 7). Secondly, for HBeAg-negative patients with a normal ALT concentration, a liver biopsy is required to determine the degree of fibrosis or necrotizing inflammation (8, 9), however, liver biopsy is not acceptable for patients without obvious symptoms. Thirdly, although an increasing number of studies have reported that the HBsAg concentration varies significantly among the phases of CHB and provide insight into the HBV life cycle, effective diagnostic parameters to evaluate the clinical application of HBsAg are still lacking (7, 8). In addition, according to previous guidelines, five indicators of HBsAg, HBeAg, HBV DNA, ALT and liver disease or even a combination of eight indicators are required to distinguish the four phases of chronic hepatitis B (5, 10). Therefore, a more applicable and simpler predictive model is needed.

Many studies have shown that two novel HBV biomarkers, including HBV RNA and HBcrAg, these biomarkers are closely related to other conventional markers, and they may be useful to distinguish the natural course of CHB and monitor disease progression (11–13). A recent study suggested that HBV RNA in serum represents pregenome RNA (pgRNA), which is partially reverse transcribed and encapsulated in virus-like particles (14). Given that pgRNA is transcribed directly from covalently closed circular DNA (cccDNA), the HBV RNA concentration may serve as a surrogate marker for transcriptionally active cccDNA (15). HBcrAg is a composite biomarker that incorporates several viral antigens expressed from the HBV pre-core/core gene, including the hepatitis B core antigen (HBcAg), HBeAg, and p22 core-related antigen. In a previous study, HBcrAg was positively correlated with the intrahepatic cccDNA concentration and the serum HBV DNA concentration (16). Some studies, as well as clinical guidelines for the treatment of CHB, have suggested that the concentrations of serum HBV RNA and HBcrAg should be used to monitor the course of CHB and to determine prognosis (10, 17, 18), but there is no exact cutoff value to distinguish the CHB phases.

In this study, we aimed to examine the distribution of serum HBV RNA and HBcrAg concentrations in CHB patients without treatment currently in different phases to explore the clinical significance of these two novel HBV biomarkers and their predictive value in distinguishing the four phases of CHB.

## Materials and methods

### Patients

A total of 189 CHB patients without treatment currently were enrolled from a cohort of 3,962 participants with CHB in Wuwei from 2018 to 2021. Four phases of CHB were distinguished according to HBV DNA and serum alanine aminotransferase (ALT), together with the HBeAg/hepatitis B e antibody status (5, 10). HBeAg-positive (e^+^) participants were categorized as immune-tolerant (IT [e^+^]) if the HBV DNA concentration was ≥2 × 10^7^ log_10_ IU/mL and the ALT concentration was normal, or as HBeAg-positive active immune hepatitis (IC [e^+^]) if the HBV DNA concentration was >2 × 10^4^ log_10_ IU/mL and the ALT concentration was elevated. HBeAg-negative (e^−^) participants were categorized as having inactive CHB (ENQ [e^−^]) if the HBV DNA concentration was ≤2 × 10^3^ log_10_ IU/mL and the ALT concentration was normal, and as HBeAg-negative reactive hepatitis (ENH [e^−^]) if the HBV DNA concentration was ≥2 × 10^3^ log_10_ IU/mL and the ALT concentration was elevated. The exclusion criteria were as follows: alcoholic liver disease; liver damage caused by drugs or alcohol; infection with hepatitis A virus, hepatitis C virus (HCV), hepatitis D virus (HDV), human immunodeficiency virus (HIV); and patients with CHB who had undergone treatment.

### Serum HBV pgRNA quantification

HBV RNA was detected using the RNA simultaneous amplification testing method based on real-time fluorescence detection of RNA transcription-mediated nucleic acid amplification (Shengxiang Biotechnology, Changsha, China). The LLOD of HBV RNA was 100 copies/mL. The non-detected HBV RNA level was set to 0 copies/mL. The results are presented as log_10_ copies/mL. HBV RNA concentrations were further categorized as ≤2, 2 to ≤4, 4 to ≤6, or >6 log_10_ copies/mL.

### Serum HBcrAg quantification

Serum HBcrAg was measured using the Lumipulse G HBcrAg chemiluminescent enzyme immunoassay (Fujirebio, Tokyo, Japan). The linear measurement range of the assay is 2–7 log_10_ U/mL. Samples with an HBcrAg concentration of >7 log_10_ U/mL were diluted with a specific dilution reagent and retested to quantify the HBcrAg concentration. The results are presented as log_10_ U/mL. HBcrAg concentrations were further categorized as <3, 3 to <4, 4 to 6.8, or ≥6.8 log_10_ U/mL.

### Conventional assays

Other conventional assays were examined in Gansu Wuwei Tumor Hospital and collected by an investigator. The serum HBV DNA concentration was measured using a hybrid capture assay (Daan Gene, Guangzhou, China) before 2019, followed by a PCR fluorescence probing assay (Abbott, USA). The LLOD of HBV DNA was 100 IU/mL. The non-detected HBV DNA level was set to 0 IU/mL. The results are presented as log_10_ IU/mL.

Liver function, alpha-fetoprotein (AFP), anti-HCV, abdominal ultrasonography, and liver stiffness measurements, as well as qualitative serological assays for HBV, were conducted at Gansu Wuwei Tumor Hospital. Liver function measurements, including measurements of aspartate aminotransferase (AST), alanine aminotransferase (ALT), total bilirubin (TBIL), gamma-glutamyl transpeptidase (GGT), and albumin (ALB) concentrations, were measured using standard laboratory procedures. A chemiluminescence analysis reagent (Roche, Manheim, Germany) was used to assess AFP. Enzyme-linked immunosorbent assay reagents (KHB, Shanghai, China) were used for the initial qualitative testing of HBsAg, HBeAg, anti-HBs, anti-HBe, anti-HBc, and anti-HCV. Abdominal ultrasonography was performed by an experienced doctor using the ACUSON S1000 Ultrasound System (Siemens Healthneers, USA). A one-dimensional transient elastography technique applied with the Fibroscan System (Echosens, Paris, France) was used to measure liver stiffness.

### Statistical analysis

The data are presented as the median and interquartile range for continuous variables and as numbers (percentage) for categorical variables. The characteristics were compared across the CHB phases using the Kruskal–Wallis test, chi-square test, or Fisher's exact test, as appropriate. Associations were tested with Spearman's rank correlation (r). A series of multinomial logistic regression models were used to test the odds of a higher ALT category vs. the lowest category by HBV RNA, HBcrAg, and HBV DNA, respectively.

Receiver operating characteristic (ROC) curves were drawn to determine the performance of each biomarker in determining the CHB phase. The biomarker with the highest area under the ROC (AUROC) curve value was further examined to identify the Youden's index according to the following equation to determine the cut-off value: sensitivity + specificity – 1. Samples are divided into the test and training sets and the decision tree model is performed using “caret” and “rpart” packages (19–21). SAS version 9.4 (SAS Institute, Cary, NC, USA) and R, version 4.1.3 (R Foundation for Statistical Computing, Vienna, Austria) was used for statistical analysis. A two-sided *P*-value of <0.05 was considered statistically significant.

## Results

### Characteristics of patients in different phases of CHB

A total of 189 CHB patients without treatment currently were divided into four phase: IT (*n* = 46), IC (*n* = 45), ENQ (*n* = 49), and ENH (*n* = 49). Of these, 91 patients (48.14%) in the IT and IC phases were HBeAg-positive, and 98 patients (51.86%) in the ENQ and ENH phases were HBeAg-negative. Patients' baseline characteristics are presented in Table 1. Significant differences existed among the different phases of CHB based on the included staging criteria. Patients in the HBeAg-positive phase were younger than those in the HBeAg-negative phase. The median ALT, AST, GGT, CAP, and TBIL concentrations were higher in the IC and ENH phases than in the IT and ENQ phases.

**Table 1:** Clinical characteristics of the patients in this study.

	**IT (*n* = 46)**	**IC (*n* = 45)**	**ENQ (*n* = 49)**	**ENH (*n* = 49)**	***P*** **-value**
Male	14 (30.4%)	25 (53.2%)	19 (41.3%)	31 (67.4%)	**/**
Age, years	29 (25, 40)	31 (24, 38)	45 (34, 53)	43 (37–52)	<0.01
ALB, g/L	48.6 (44.5, 50.2)	48.3 (45.1, 50.2)	48.6 (47.0, 50.4)	49.0 (46.8, 52.2)	0.13
TBIL, umol/L	16.0 (11.9, 20.4)	16.5 (13.7, 21.9)	16.0 (12.1, 20.4)	18.1 (14.1, 21.9)	0.31
LSM	4.5 (4.0, 5.3)	5.3 (4.6, 7.6)	4.3 (3.5, 5.1)	5.3 (4.5, 6.4)	<0.01
CAP	221 (194, 240)	219 (179, 237)	238 (214, 268)	235 (207, 293)	0.02
AFP	2.2 (1.1, 2.8)	2.9 (2.2, 4.9)	2.2 (1.3, 2.8)	2.7 (2.2, 3.9)	0.24
GGT	15.7 (11.5, 21.2)	31.1 (18.5, 56.2)	18.0 (13.0, 24.5)	33.0 (19.0, 44.0)	<0.01
ALT, IU/L	24.5 (18.5, 29.8)	62.0 (49.0, 130.6)	25.6 (19.0, 29.5)	62.7 (47.6, 88.5)	<0.01
AST, IU/L	22.4 (18.2, 27.0)	42.3 (34.4, 69.5)	23.8 (20.0, 27.6)	39.4 (32.4, 49.8)	<0.01
HBV DNA, log_10_ IU/mL	7.96 (7.74, 8.00)	7.78 (7.05, 7.97)	2.09 (2.00, 2.78)	4.06 (3.64, 4.77)	<0.01
HBV DNA quantifiable, *n* (%)	45 (100.0)	46 (100.0)	26 (53.1)	49 (100.0)	<0.01
HBV RNA, Log_10_ copies/mL	6.73 (6.46, 6.96)	6.63 (5.90, 7.02)	0.00 (0.00, 1.14)	2.67 (1.23, 3.47)	<0.01
HBV RNA quantifiable, *n* (%)	45 (100.0)	46 (100.0)	7 (14.3)	30 (61.2)	<0.01
HBcrAg log_10_ U/mL	8.60 (8.50, 8.80)	8.50 (7.90, 8.70)	2.70 (2.00, 3.10)	3.30 (2.80, 4.40)	<0.01
HBcrAg quantifiable, *n* (%)	45 (100.0)	46 (100.0)	15 (30.6)	29 (59.2)	<0.01

IT, immune tolerance; IC, immune clearance; ENQ, hepatitis B e antigen-negative inactive/quiescent carrier phase; ENH, hepatitis B e antigen-negative hepatitis.

### Serum HBV RNA and HBcrAg concentrations across different CHB phases

**Figure 1:**
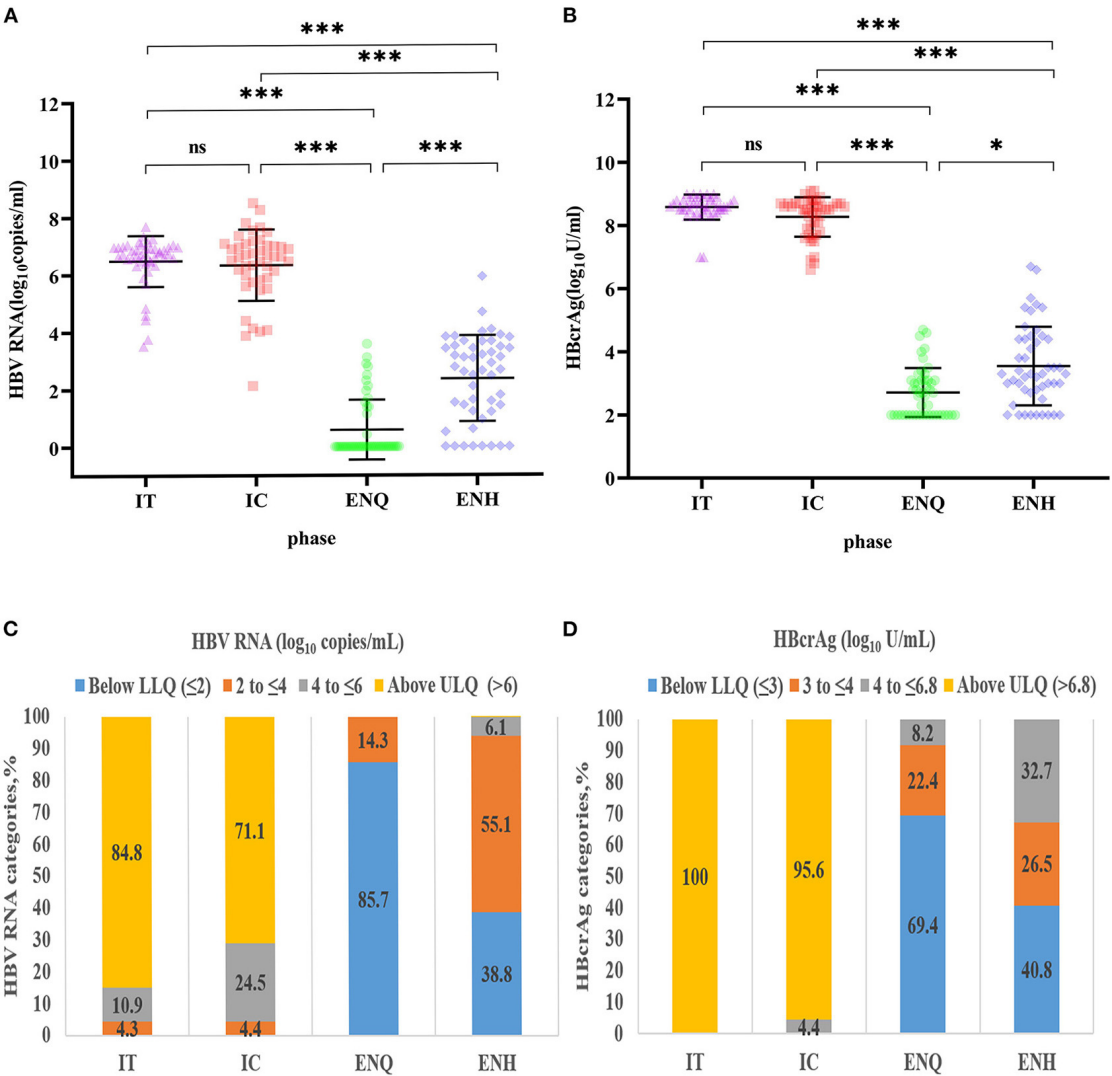
Distribution of HBV RNA and HBcrAg concentrations during the natural course of CHB infection. **(A)** HBV RNA concentration distribution during the natural course of CHB infection. ***p < 0.001; *p < 0.05; ns, no statistical significance. **(B)** HBcrAg concentration distribution during the natural course of CHB infection. ***p < 0.001; *p < 0.05; ns, no statistical significance. **(C)** HBV RNA (log_10_ copies/mL) category distribution during the natural course of CHB infection. **(D)** HBcrAg (log_10_ IU/mL) category distribution during the natural course of CHB infection. IT, immune tolerance; IC, immune clearance; ENQ, hepatitis B e antigen-negative inactive/quiescent carrier phase; ENH, hepatitis B e antigen-negative hepatitis; HBV, hepatitis B virus; HBcrAg, hepatitis B core-related antigen; CHB, chronic hepatitis B.

The differences in HBV RNA concentration among the four phases are depicted in Figure 1. Except for the IT phase and the IC phase, the HBV RNA concentration varied significantly between each phase of CHB infection (Figure 1A). IT (6.73, 6.46–6.96) and IC (6.63, 5.90–7.02) have a similar concentration of HBV RNA, followed by ENH (2.67, 1.23–3.47) and ENQ (0.00, 0.00–1.14). When dividing the HBV RNA into ≤2, 2 to ≤4, 4 to ≤6, >6, 84.8% in the IT phase and 71.1% in the IC phase had a concentration of >6 log_10_ copies/mL. While 85.7% in the IT phase and 40.8% in the IC phase had a concentration of ≤2 log_10_ copies/mL (Figure 1C). The HBcrAg concentration distribution in the four phases was similar to that of HBV RNA (Figures 1B,D).

### Associations of HBV RNA and HBcrAg concentrations with ALT and HBV DNA

**Figure 2:**
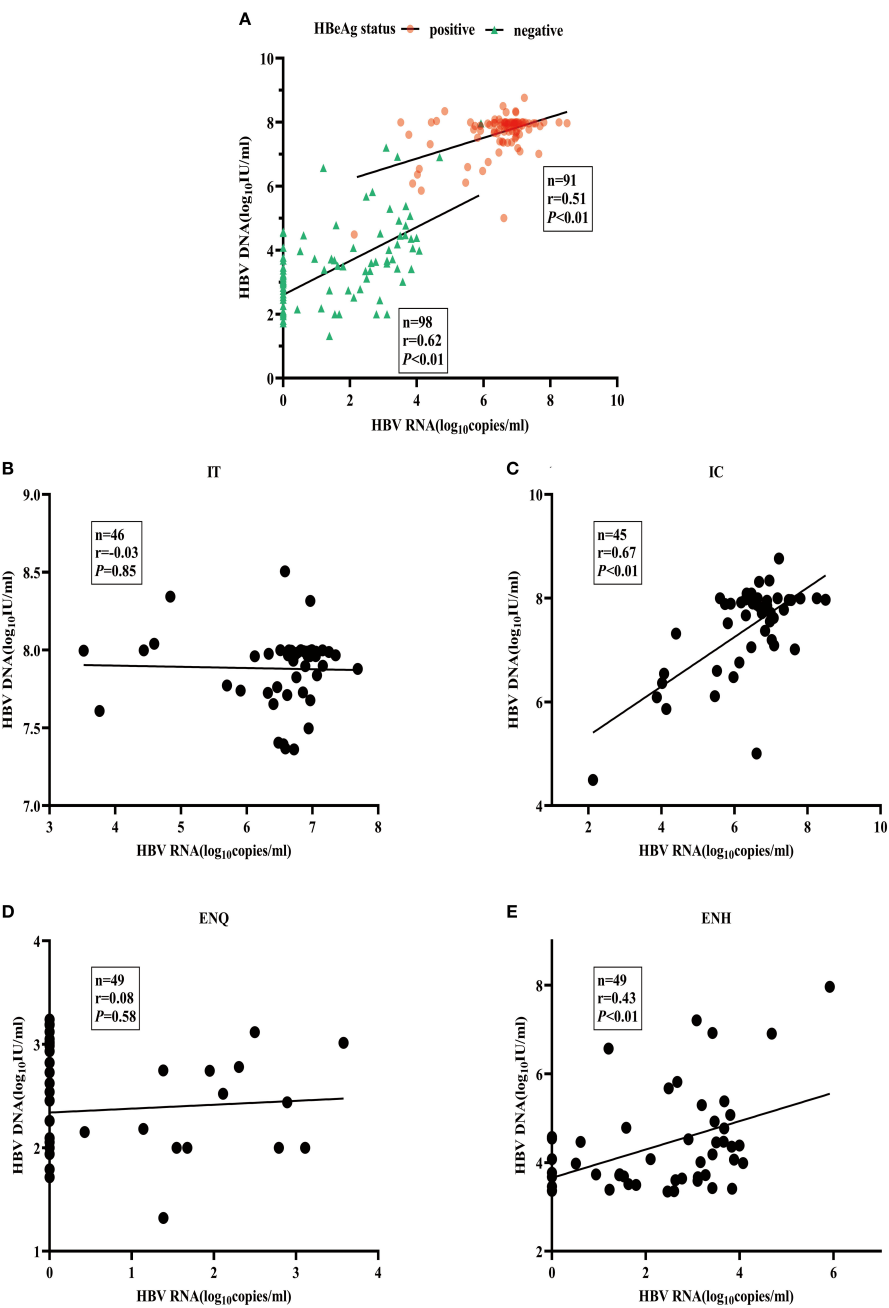
Association between serum HBV RNA and HBV DNA. **(A)** HBV RNA and HBV DNA by HBeAg status. **(B)** HBV RNA and HBV DNA in the IT phase. **(C)** HBV RNA and HBV DNA in the IC phase. **(D)** HBV RNA and HBV DNA in the ENQ phase. **(E)** HBV RNA and HBV DNA in the ENH phase.

The heatmap shows a strong correlation between HBcrAg, and HBV RNA, as well as between the ALT and HBV DNA concentrations (Supplementary Figure 1). The median HBV DNA concentration was consistently 1–2 logs higher than the HBV RNA concentration throughout the natural course of CHB infection (Supplementary Figure 2). The association between HBV RNA and HBV DNA was different in patients with HBeAg-positive patients (r = 0.51) and HBeAg-negative patients (r = 0.62) (Figure 2A). When further divided into four phases, the pattern of association varied significantly. A strong relationship between concentrations was observed in the IC and ENH phases (r = 0.67, and r = 0.43, respectively) (Figures 2C,E), but not in the IT and ENQ phases (r = −0.03, and r = 0.08, respectively) (Figures 2B,D). The HBcrAg and HBV RNA concentrations were similarly associated with HBV DNA, but unlike HBV RNA, HBcrAg was more strongly associated with HBV DNA in the ENH phase (r = 0.85, *P* < 0.01) (Supplementary Figures 3A–E).

We stratified ALT using the upper limit of normal (40 IU/L) as the standard and the sum of the test values and 40 as the ratio of ≤1, 1 to 2, and >2, respectively. Among HBeAg-negative patients, HBV RNA, HBcrAg, and HBV DNA concentrations were significantly associated with the ALT category (*P* < 0.01) (Supplementary Figures 4A–C). In HBeAg-positive patients, the HBV RNA concentration was not associated with ALT and the ALT categories (*P* = 0.39) (Supplementary Figure 4A), and the HBcrAg concentration was not associated with the ALT category (*P* = 0.99) (Supplementary Figure 4D). There was a weak positive association (*P* = 0.02) between the HBV DNA concentration and the ALT category (Supplementary Figure 4B). All *P* values are presented in Supplementary Tables 1, 2.

The serum HBV RNA concentration and the RNA/DNA ratio in HBeAg-positive patients were significantly higher than in HBeAg-negative patients (Supplementary Figure 5A). The stratified analysis showed that the ENQ phase had the lowest serum HBV RNA concentration and the lowest RNA/DNA ratio (IT = 0.85, IC = 0.86, ENQ = 0.00, ENH = 0.60), which were only significantly lower than in the IT and IC phases (Supplementary Figure 5B).

### Predictive value of HBV RNA and HBcrAg for distinguishing the phases of CHB

**Figure 3:**
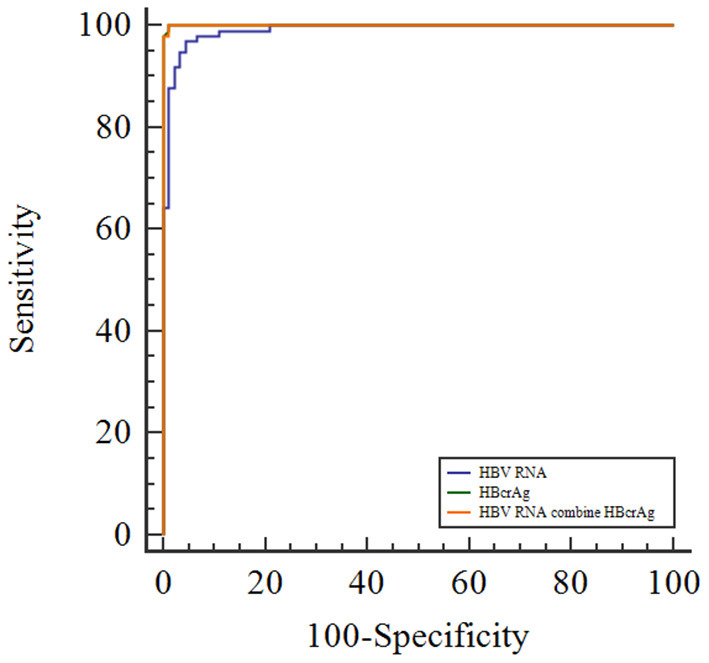
Receiver operating characteristic curve showing the diagnostic value of serum HBV RNA and HBcrAg concentrations in HBeAg-positive patients with CHB infection.

To evaluate the efficacy of HBV RNA and HBcrAg concentrations in distinguishing the HBeAg status, we employed the ROC curve analysis to calculate the AUROC curve values for distinguishing the HBeAg(+) and HBeAg(−) status was 0.991 and 1.000. The combination shows a better predictive value of 1.000. The AUROC curve values for HBV RNA and HBcrAg at cutoff values of 6.32 log_10_ copies/mL and 7.50 log_10_ IU/mL, respectively (Figure 3, Table 2).

**Table 2:** Receiver operating characteristic curve showing the diagnostic value of serum HBV RNA and HBcrAg level for HBeAg-positive status in chronic HBV infection.

**Test result variables**	**AUC (95% CI)**	**Cutoff value**	**Accuracy (%)**	***P*** **-value**	**SEN (%)**	**SPE (%)**	**PPV (%)**	**NPV (%)**
HBV RNA	0.991 (0.981, 1.000)	6.32	96.3	<0.001	96.94	95.60	95.96	96.67
HBcrAg	1.000 (0.999, 1.000)	7.50	99.5	<0.001	100.00	98.90	98.99	100.00
HBV RNA combine HBcrAg	1.000 (0.999, 1.000)	–	99.5	<0.001	100.00	98.90	98.99	100.00

AUC, area under; NPV, negative predictive value; PPV, positive predictive value; ROC, receiver operating characteristic curve; SEN, sensitivity; SPE, specificity.

Although HBV RNA and HBcrAg concentrations tended to decrease during progression to the ENQ and ENH phases of CHB, the concentrations increased when HBV was reactivated. We observed significant differences in the serum HBV RNA and HBcrAg concentrations in HBeAg-negative status in previous studies. But the above results suggest that the ability of these novel HBV biomarkers to distinguish ENQ and ENH independently or jointly was not outstanding. We examined the value of serum HBV RNA and HBcrAg in identifying the ENQ and ENH phases of CHB. With cutoff values of 4.01 log_10_ U/mL and 4.50 log_10_ IU/mL, the AUROC curve values for HBV RNA and HBcrAg were 0.825 and 0.709 for distinguishing the ENQ and ENH phases, respectively. The AUROC curve value for the combination of HBV RNA and HBcrAg to distinguish the ENQ and ENH phases was 0.836 (Supplementary Figure 6, Supplementary Table 3). Therefore, we examined whether these novel biomarkers can distinguish between the ENQ and ENH phases, or even between the whole natural course of CHB when used in combination with conventional markers.

**Figure 4:**
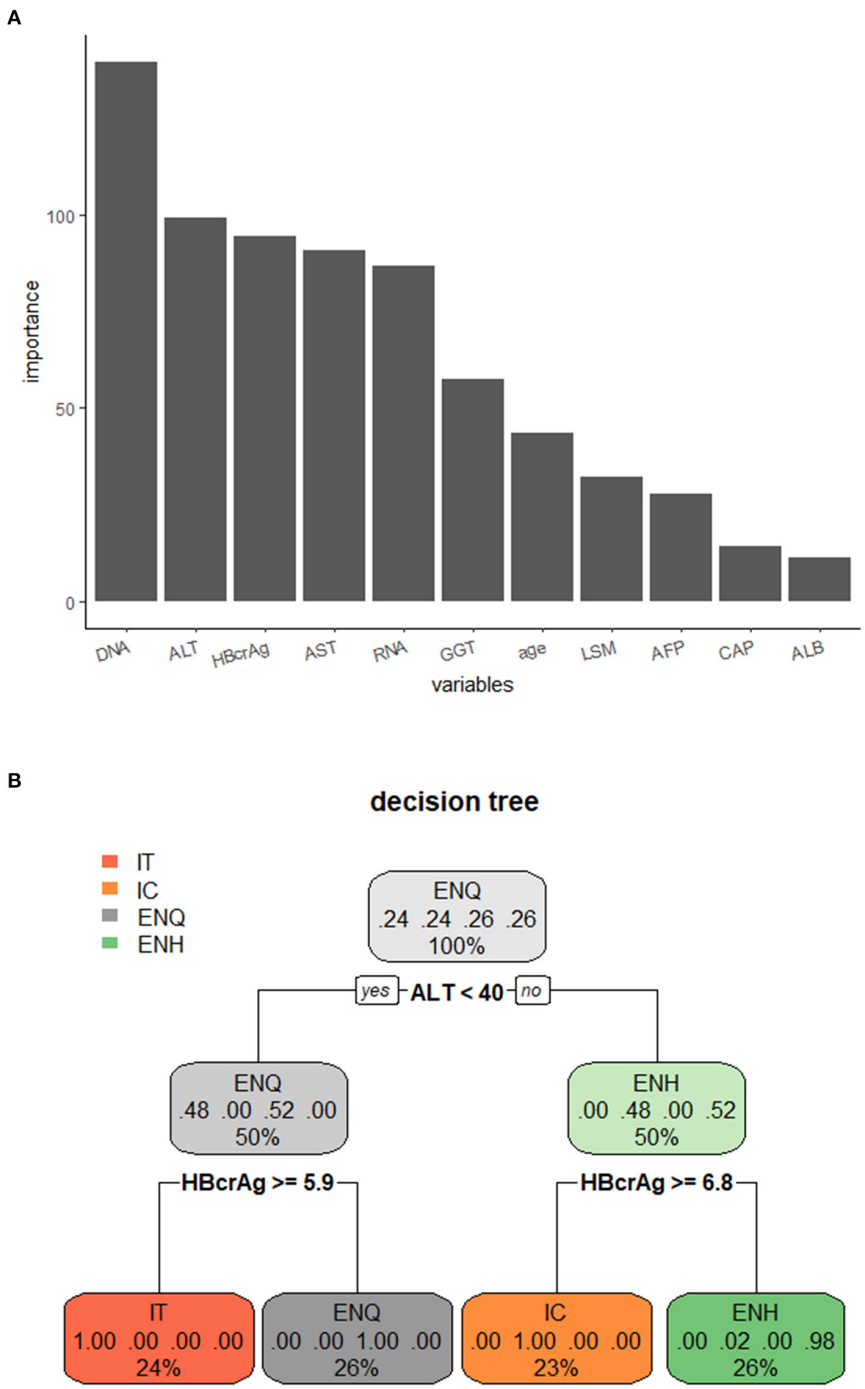
Decision tree model for distinguishing the phases of the natural course of CHB infection. **(A)** Importance of the variables in the decision tree model. **(B)** Decision tree model for distinguishing the phases of the natural course of CHB infection using ALT combined with HBcrAg. IT, immune tolerance; IC, immune clearance; ENQ, hepatitis B e antigen-negative inactive/quiescent carrier phase; ENH, hepatitis B e antigen-negative hepatitis.

The samples were divided into the training set (75%) and the test set (25%). The important parameters for use in the decision tree model were identified; ALT and HBV DNA were the most important factors (Figure 4A). The decision tree model with a criterion of ALT < 40 IU/L and HBcrAg ≥ 5.9 log_10_ IU/mL or HBcrAg ≥ 6.8 log_10_ IU/mL. We observed the prediction accuracy of the decision tree model based on ALT with HBcrAg in the four phases was the best, at 95.65%, and ALT with HBV RNA was 93.55% (Figure 4B, Supplementary Figure 7, Supplementary Table 4).

## Discussion

In this study of 189 CHB patients without treatment currently from Northwest China, we investigated the concentrations of HBV RNA and HBcrAg were highest in the IT and IC phases. HBV RNA and HBcrAg correlated with HBV DNA in the HBeAg^+^ and HBeAg^−^ status, but their association with HBV DNA differed among phases. The decision tree model using a combination of HBcrAg and ALT is the best combination to differentiate the four phases.

The present study demonstrated that HBV RNA and HBcrAg concentrations displayed a wide distribution, with a higher concentration in the HBeAg-positive status than in the HBeAg-negative status. HBV RNA and HBcrAg concentrations varied significantly among the natural phases of CHB infection. The concentrations were highest in the IT phase, followed by the IC, ENH, and ENQ phases successively. These findings are in line with previous reports (17, 18, 22).

Regardless of the phase of CHB, the median HBV DNA concentration was consistently 1–2 log_10_ higher than the HBV RNA concentration throughout the natural course of CHB infection (12, 22–24). However, other studies have shown that the median HBV DNA concentration in patients with CHB after antiviral therapy is higher than the concentration of HBV RNA. Moreover, when HBV DNA is below the detection limit, HBV RNA can still be detected in some patients (25, 26). Regardless of the status of HBeAg negativity, the HBV DNA concentration was significantly associated with the ALT concentration. However, the HBV RNA and HBcrAg concentrations were associated with the ALT concentration only in HBeAg-negative patients, suggesting that changes in HBV RNA and HBcrAg concentrations were inconsistent with changes in the HBV DNA concentration in the setting of ALT changes (11). These results indicate that HBV DNA is sensitive to antiviral drugs, and HBV DNA may fall below the detection limit after administration, preventing the natural course of CHB from being distinguished. Compared with HBV DNA, the HBV RNA and HBcrAg may be irreplaceable in distinguishing the natural course of CHB, but further research is needed to prove this speculation.

During the natural history of chronic HBV infection, the serum HBV RNA concentration and the HBV RNA to DNA ratio changed significantly. The serum HBV RNA concentration and the ratio of RNA to DNA were higher in HBeAg-positive patients than in HBeAg-negative patients. The stratified analysis revealed that the serum HBV RNA concentration and the ratio of RNA to DNA in the ENQ phase were lower than in the other three phases. This finding is similar to that of a recently published study showing a significantly higher proportion of HBeAg-negative ENH phases than HBeAg-negative ENQ phases (1.04 vs. 0.85; *P* = 0.01) (22). The lower limit of detection (LLOD) for serum HBV DNA and RNA in this study was 500 and 100 copies/mL, respectively. However, a previously published study observed contradicting results, in which the ratio in the HBeAg-negative ENH phase was remarkably similar to that in the HBeAg-negative ENQ phase (0.53 vs. 0.53; *P* = 0.71) (12). The LLOD of HBV RNA was 1.65 log_10_ U/mL, and that of HBV-DNA was 1 log_10_ IU/ mL. In the present study, the LLOD of HBV RNA was 0 copies/mL, and the HBV DNA was 100 IU/mL. These results demonstrate that the virus exhibited different replication levels over the natural course of CHB infection. Previous studies on the changing patterns of HBsAg, which is another alternative biomarker of intrahepatic cccDNA transcription activity, over the natural course of CHB infection, also indicated that HBV exhibited different replication levels throughout the natural course of CHB infection (27, 28). However, the difference in the LLOD of HBV RNA and HBV DNA in these studies will affect the calculated HBV RNA to DNA ratio, so these studies cannot be directly compared, which highlights the importance of developing standardized test methods in the future.

In this study, we documented a strong overall association of HBV RNA and HBcrAg with HBV DNA, suggesting that quantitative HBV RNA and HBcrAg may partly reflect viral replication. This was most evident in the IC and ENH phases, which are active states of HBV replication. However, we did not observe an association between the IT and ENQ phases for HBV RNA, HBcrAg, or HBV DNA, which is not consistent with one Asian and one European study (17, 18). This may be due to the different genotypes of the patients studied. In our previous study, we found that the main HBV genotype in the Wuwei region was (C/D) (29), while the genotypes in two other studies were primarily (B/C) and (A/D) (17, 18). Therefore, we need to further explore the relationship between novel biomarkers and conventional markers in different genotypes in different phases of the natural history of chronic HBV infection.

In this study, HBV RNA and HBcrAg, both alone and in combination, demonstrated high predictive value for distinguishing HBeAg(+) and HBeAg(–) status. However, the predictive value was lower for distinguishing ENQ and ENH phases, with an AUROC curve value of 0.825 for HBV RNA, 0.709 for HBcrAg, and 0.836 for combination. This is similar to the AUROC (0.833) of HBV RNA in a previous study, but another reported a much higher AUROC (0.931) of HBcrAg than ours (22, 30). One possible reason for this difference may be that the number of patients in the ENQ phase in our study was small, resulting in a decrease in the discrimination ability.

In this study, the decision tree prediction model based on the combination of novel HBV biomarkers and conventional markers could accurately distinguish the natural course of CHB, and the accuracy of HBV RNA and HBcrAg combined with ALT to distinguish the four phases of chronic HBV infection was 93.48% and 95.65%, respectively. However, when used in combination with HBV DNA, the predictive effect did not increase significantly, which may have been because HBV RNA and HBcrAg can replace the predictive value of HBV DNA in the four phases of CHB infection. In previous studies, the decision tree model has been used to distinguish CHB, cirrhosis, and hepatocellular carcinoma (31). These results suggest that novel HBV biomarkers can accurately differentiate the four phases of CHB infection in place of HBV DNA. This study has two main limitations that should be noted. First, given the shortcomings of retrospective studies, further prospective studies are needed to validate the prospective use of HBV RNA and HBcrAg for distinguishing the history of CHB infection in clinical practice. Second, it is necessary to recruit more patients or to obtain multi-center data to support our findings.

## Conclusion

In conclusion, this study demonstrated the concentrations of HBV RNA and HBcrAg were highest in the IT and IC phases. HBV RNA and HBcrAg were positively related to conventional markers, but the relationship was varied among four phases These novel biomarkers combined with conventional biomarkers could accurately distinguish the different phases of the natural course of CHB infection. Thus, they might be useful as serological markers to detect potential HBV infection. Further elucidation of the clinical significance of serum HBV RNA and HBcrAg concentrations will contribute greatly to the clinical management of patients with HBV infection in the future.

## Data availability statement

The raw data supporting the conclusions of this article will be made available by the authors, without undue reservation.

## Ethics statement

The studies involving human participants were reviewed and approved by Air Force Medical University Ethics Committee. The patients/participants provided their written informed consent to participate in this study.

## Author contributions

ZJ, ZS, and YY designed the study and reviewed and revised the manuscript. WW and XY coded and analyzed the data. WW, XY, and WeiZ wrote the manuscript. HZ, XK, ZH, and TF collected data. WenZ, WJ, CL, HT, and FW helped interpret the data. All authors read and approved the final manuscript.

## Funding

This study was supported by the China Special Grant for the Prevention and Control of Infection Diseases (no. 2017ZX10105011).

## Conflict of interest

The authors declare that the research was conducted in the absence of any commercial or financial relationships that could be construed as a potential conflict of interest.

## Publisher's note

All claims expressed in this article are solely those of the authors and do not necessarily represent those of their affiliated organizations, or those of the publisher, the editors and the reviewers. Any product that may be evaluated in this article, or claim that may be made by its manufacturer, is not guaranteed or endorsed by the publisher.

## Abbreviations

AUROC: area under the receiver operating characteristic curve
cccDNA: covalently closed circular DNA
CHB: chronic hepatitis B virus infection
ENQ: hepatitis B e antigen-negative inactive/quiescent carrier phase
ENH: hepatitis B e antigen-negative hepatitis
HBcrAg: hepatitis B core-related anti-gen
HBeAg: hepatitis B e antigen
HBsAg: hepatitis B surface antigen
HBV: hepatitis B virus
HCC: hepatocellular carcinoma
HCV: hepatitis C virus
HDV: hepatitis D virus
HIV: human immunodeficiency virus
IT: immune tolerance
IC: immune clearance
PCR: polymerase chain reaction
WHO: World Health Organization.
