# Supplementary material for: Clinical significance of novel biomarkers to predict the natural course of hepatitis B infection
Source: Front Public Health. 2022 Oct 28;10:1037508. doi: 10.3389/fpubh.2022.1037508 (PMC9650535; doi:10.3389/fpubh.2022.1037508)
Supplement: Supplementary file 1 [file Data_Sheet_1.docx]

Supplementary Material

**Supplementary Figure 1** Associations between HBV RNA and HBcrAg levels and Conventional markers.

**Supplementary Figure 2** The median level of each biomarker during the natural phases of CHB infection.

**Supplementary Figure 3** Association between serum HBcrAg and HBV DNA levels in various phases of CHB.

**Supplementary Figure 4** HBV RNA (log_10_ copies/mL), HBcrAg (log_10_ U/mL), and HBV DNA (log_10_ IU/mL) by ALT (ULN) categories.

**Supplementary Figure 5** The ratio of serum HBV RNA to HBV DNA in various groups.

**Supplementary Figure 6** Receiver operating characteristic curve showing the diagnostic value of serum HBV RNA and HBcrAg level for ENQ phase and ENH phase.

**Supplementary Figure 7** Decision tree model for distinguishing different phases of the natural course of CHB infection using ALT combined with HBV RNA.

**Supplementary Table 1** Associations between HBV RNA, HBcrAg, and HBV DNA, respectively, with ALT categories, among HBeAg positive participants.

**Supplementary Table 2** Associations between HBV RNA, HBcrAg, and HBV DNA, respectively, with ALT categories, among HBeAg negative participants.

**Supplementary Table 3** Receiver operating characteristic curve showing the diagnostic value of serum HBV RNA and HBcrAg level for ENQ phase and ENH phase.

**Supplementary Table 4** Predictive values of HBV RNA and HBcrAg combine Conventional markers for distinguishing four phases of CHB.


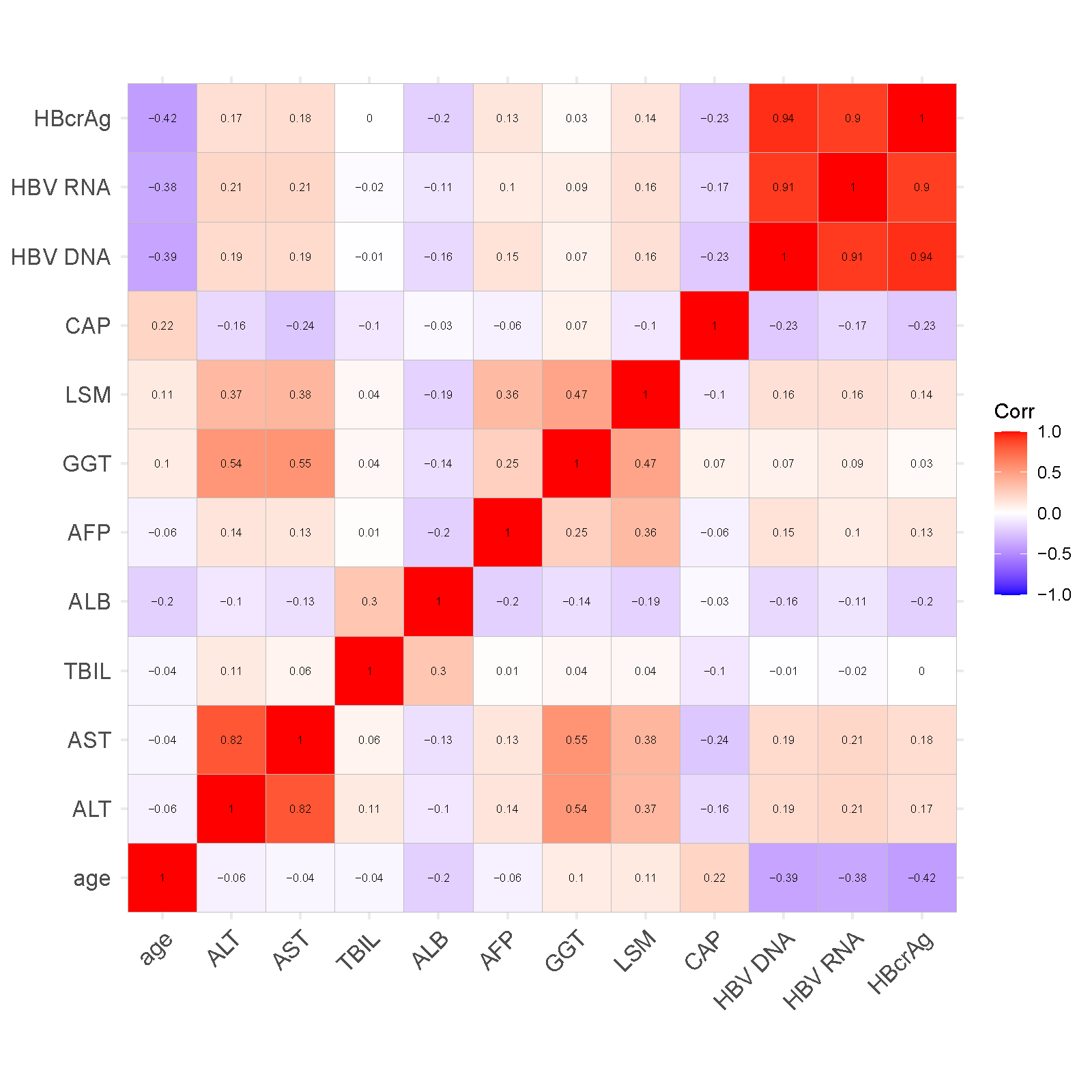


**Supplementary Figure 1. Associations between HBV RNA and HBcrAg levels and Conventional markers.**


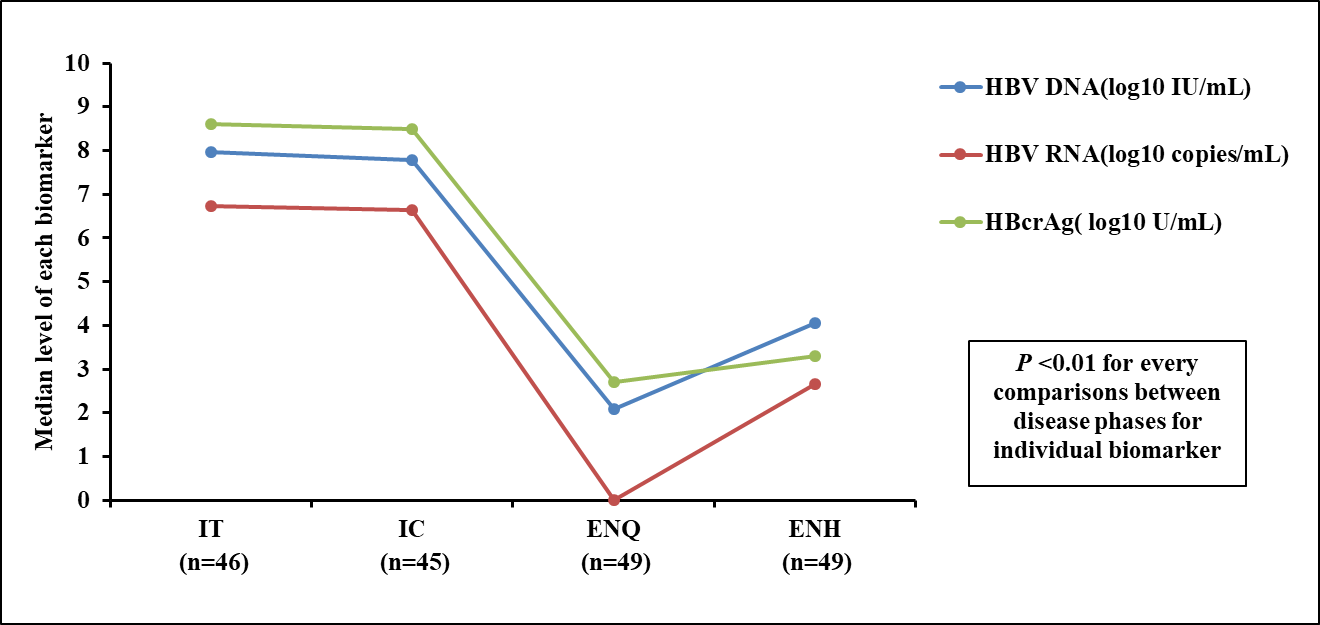


**Supplementary Figure 2. The median level of each biomarker during the natural phases of CHB infection.** IT, immune tolerance; IC, immune clearance; ENQ, hepatitis B e antigen-negative inactive/quiescent carrier phase; ENH, hepatitis B e antigen-negative hepatitis; HBV, hepatitis B virus; HBcrAg, hepatitis B core-related antigen; CHB, chronic hepatitis B.

**
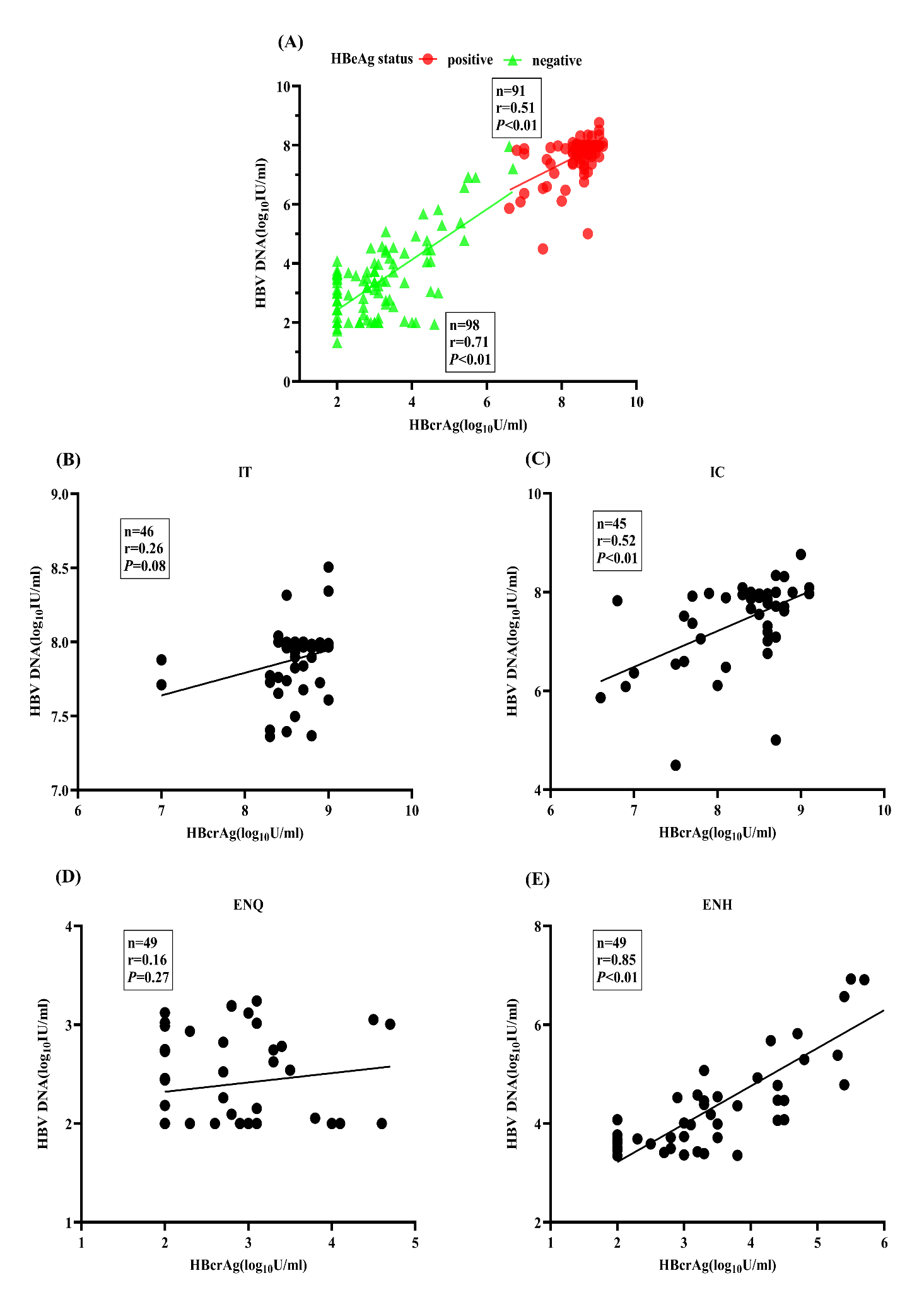
**

**Supplementary Figure 3. Association between serum HBcrAg and HBV DNA levels in various phases of CHB.** (A) HBcrAg and HBV DNA by HBeAg status. (B) HBcrAg and HBV DNA in IT phase. (C) HBcrAg and HBV DNA in IC phase. (D) HBcrAg and HBV DNA in ENQ phase. (E) HBcrAg and HBV DNA in ENH phase. IT, immune tolerance; IC, immune clearance; ENQ, hepatitis B e antigen-negative inactive/quiescent carrier phase; ENH, hepatitis B e antigen-negative hepatitis; HBV, hepatitis B virus; HBcrAg, hepatitis B core-related antigen; CHB, chronic hepatitis B.

**
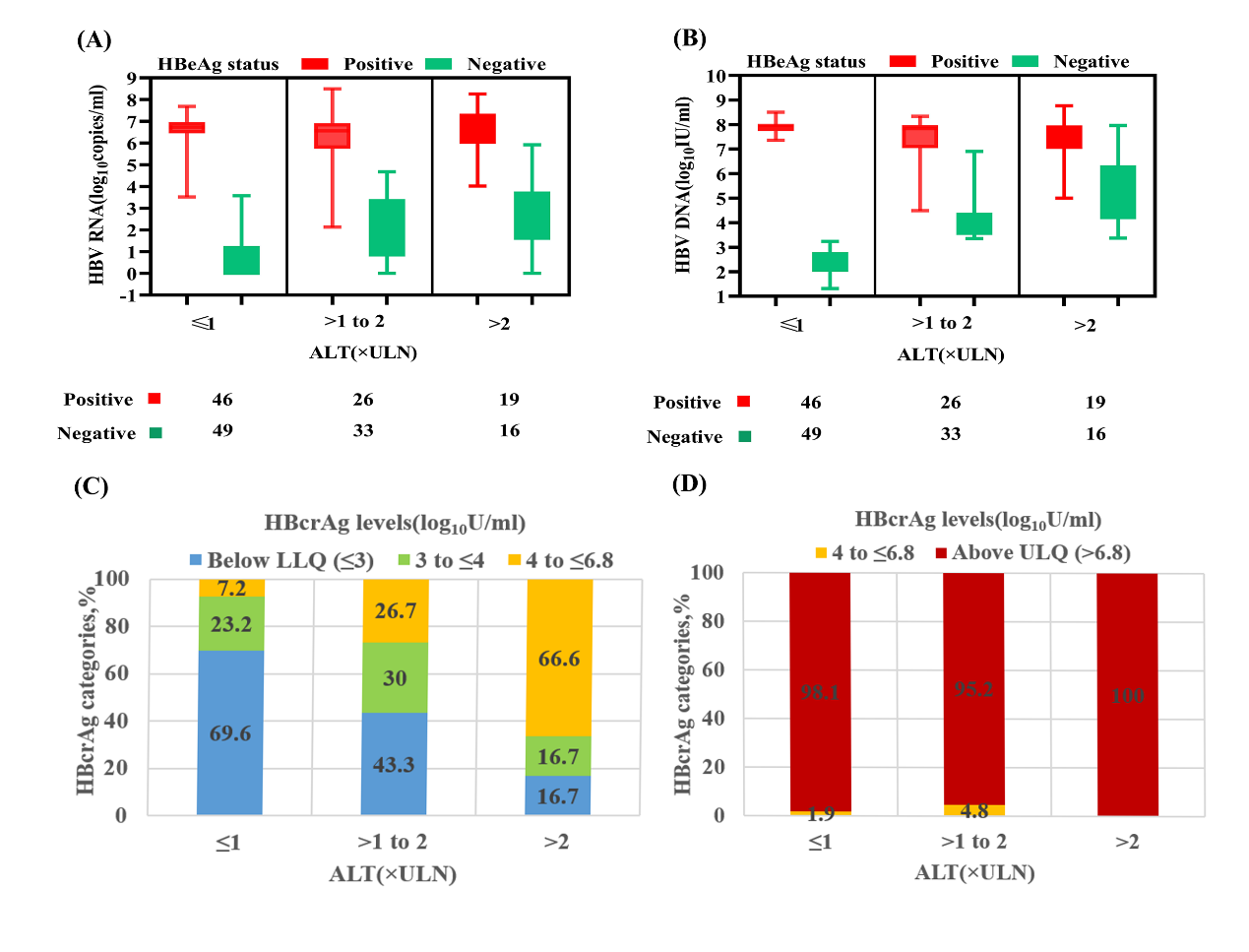
**

**Supplementary Figure 4. HBV RNA (log_10_ copies/mL), HBcrAg (log_10_ U/mL), and HBV DNA (log_10_ IU/mL) by ALT (ULN) categories.** (A) HBV RNA (log_10_ copies/mL) categories by ALT categories by HBeAg status. (B) HBV DNA (log_10_ IU/mL) categories by ALT categories by HBeAg status. (C) HBcrAg (log_10_ U/mL) categories by ALT categories in HBeAg-negative status. (D) HBcrAg (log_10_ U/mL) categories by ALT categories in HBeAg-positive status.


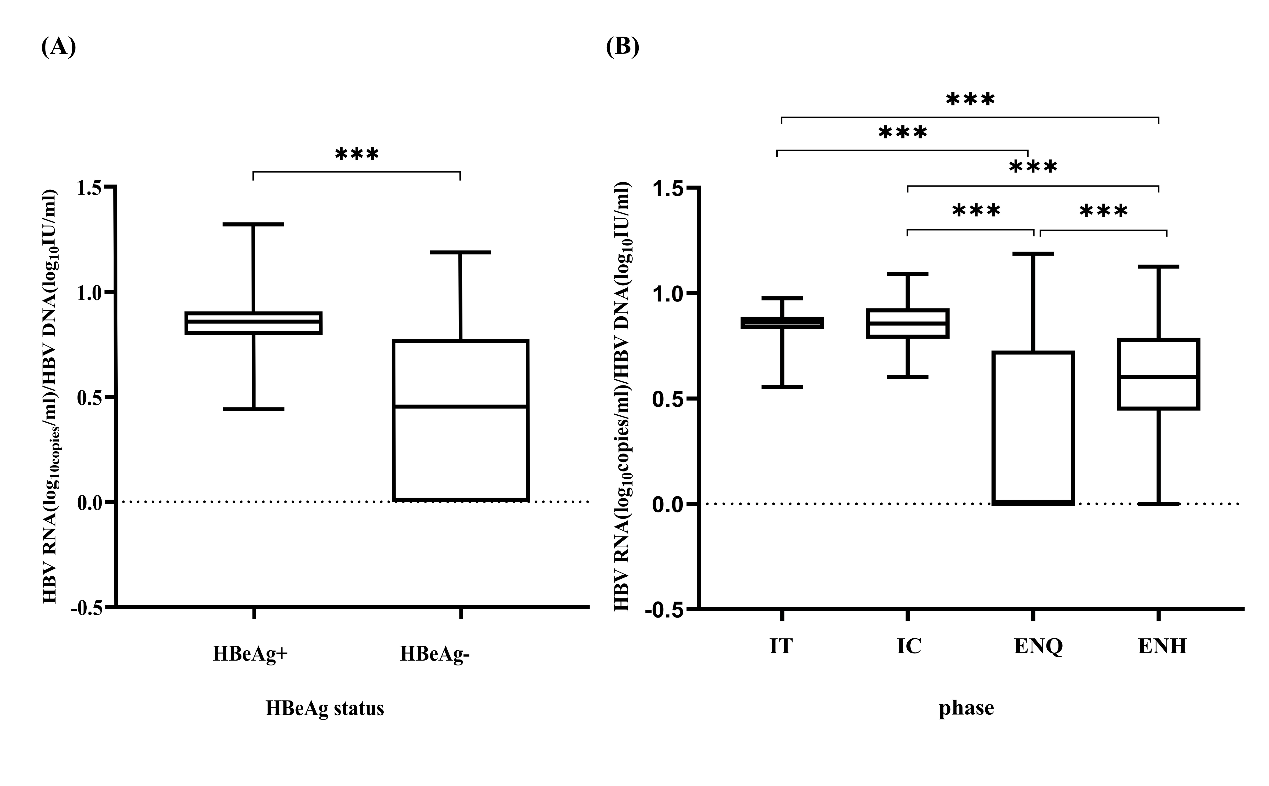


**Supplementary Figure 5. The ratio of serum HBV RNA to HBV DNA in various groups.** (A) The ratio of serum HBV RNA to HBV DNA by HBeAg status. ***, *P*<0.001. (B) The ratio of serum HBV RNA to HBV DNA in four phases of CHB. ***, *P*<0.001. IT, immune tolerance; IC, immune clearance; ENQ, hepatitis B e antigen-negative inactive/quiescent carrier phase; ENH, hepatitis B e antigen-negative hepatitis; HBV, hepatitis B virus; CHB, chronic hepatitis B.


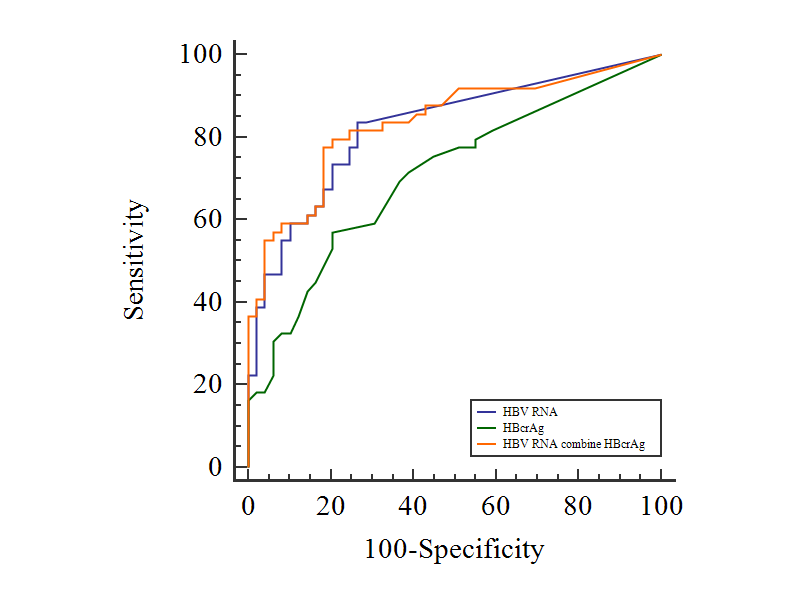


**Supplementary Figure 6. Receiver operating characteristic curve showing the diagnostic value of serum HBV RNA and HBcrAg level for ENQ phase and ENH phase.** AUC, area under curve; NPV, negative predictive value; PPV, positive predictive value; ROC, receiver operating characteristic curve; SEN, sensitivity; SPE, specificity; ENQ, hepatitis B e antigen-negative inactive/quiescent carrier phase; ENH, hepatitis B e antigen-negative hepatitis.


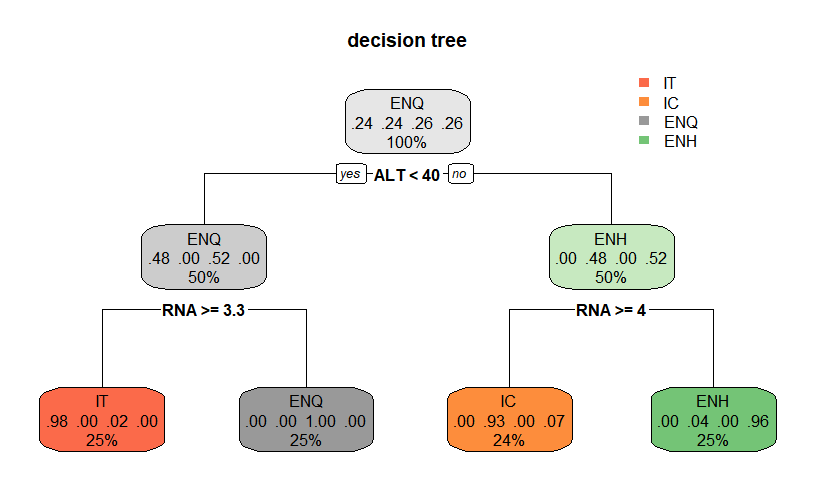


**Supplementary Figure 7. Decision tree model for distinguishing different phases of the natural course of CHB infection using ALT combined with HBV RNA.** IT, immune tolerance; IC, immune clearance; ENQ, hepatitis B e antigen-negative inactive/quiescent carrier phase; ENH, hepatitis B e antigen-negative hepatitis; CHB, chronic hepatitis B.

**Supplementary Table 1. Associations between HBV RNA, HBcrAg, and HBV DNA, respectively, with ALT categories, among HBeAg positive participants.**

|  | ALT categories | |
| --- | --- | --- |
|  | OR (95% CI) | *P*-value |
| HBV RNA - per log_10_ copies/mL |  | 0.39 |
| 2 vs 1 | 0.774(0.501 - 1.194) |  |
| 3 vs 1 | 1.093(0.622 - 1.922) |  |
| HBV DNA-per log_10_ IU/mL |  | 0.02 |
| 2 vs 1 | 0.199(0.062 - 0.634) |  |
| 3 vs 1 | 0.190(0.058 - 0.625) |  |
| HBcrAg (≥6.8 vs 4 - <6.8 log_10_ U/mL) |  | 0.99 |
| 2 vs 1 | - | - |
| 3 vs 1 | - | - |

*ALT (ULN) categories: 1: ≤1.0; 2: 1.0 - 2.0; 3: >2.0

- Unable to calculate because sample size was too small.

**Supplementary Table 2. Associations between HBV RNA, HBcrAg, and HBV DNA, respectively, with ALT categories, among HBeAg negative participants.**

|  | ALT categories | |
| --- | --- | --- |
|  | OR (95% CI) | *P*-value |
| HBV RNA - per log_10_ copies/mL |  | <0.01 |
| 2 vs 1 | 2.382(1.621 - 3.500) |  |
| 3 vs 1 | 3.159(1.921 - 5.195) |  |
| HBV DNA - per log_10_ IU/mL |  | <0.01 |
| 2 vs 1 | - | - |
| 3 vs 1 | - | - |
| HBcrAg 3 - <4 vs <3 log_10_ U/mL) |  | 0.01 |
| 2 vs 1 | 2.125(0.762 - 5.925) |  |
| 3 vs 1 | 1.545(0.248 - 9.619) |  |
| HBcrAg 4 - <6.8 vs <3 log_10_ U/mL) |  |  |
| 2 vs 1 | 3.188(0.788 - 12.897) |  |
| 3 vs 1 | 21.250(4.488 - 100.615) |  |

*ALT (ULN) categories: 1: ≤1.0; 2: 1.0 - 2.0; 3: >2.0

- Unable to calculate because sample size was too small.

**Supplementary Table 3. Receiver operating characteristic curve showing the diagnostic value of serum HBV RNA and HBcrAg level for ENQ phase and ENH phase.**

| Test result variables | AUC(95%CI) | Accuracy  (%) | *P*-value | SEN  (%) | SPE  (%) | PPV (%) | NPV  (%) |
| --- | --- | --- | --- | --- | --- | --- | --- |
| HBV RNA | 0.825  (0.735,0.894) | 78.57 | <0.001 | 83.67 | 73.47 | 81.82 | 75.93 |
| HBcrAg | 0.709  (0.608,0.796) | 68.37 | <0.001 | 57.14 | 79.59 | 73.68 | 65.00 |
| HBV RNA combine HBcrAg | 0.836  (0.748,0.903) | 79.59 | <0.001 | 80.61 | 78.57 | 80.22 | 798.01 |

AUC, area under; NPV, negative predictive value; PPV, positive predictive value; ROC, receiver operating characteristic curve; SEN, sensitivity; SPE, specificity; ENQ, hepatitis B e antigen-negative inactive/quiescent carrier phase; ENH, hepatitis B e antigen-negative hepatitis.

**Supplementary Table 4. Predictive values of HBV RNA and HBcrAg combine Conventional markers for distinguishing four phases of CHB.**

| Test result variables | Accuracy  (%) | Kappa  (%) | SEN (%) | SPE (%) | PPV (%) | NPV (%) |
| --- | --- | --- | --- | --- | --- | --- |
| ALT+HBcrAg | 95.65 | 94.20 | 95.83 | 95.55 | 95.83 | 98.55 |
| ALT+ RNA | 93.55 | 91.31 | 93.75 | 97.84 | 93.88 | 97.86 |
| ALT+HBV DNA+HBcrAg | 97.86 | 97.14 | 96.87 | 96.65 | 96.43 | 98.75 |
| ALT+HBV DNA+ RNA | 94.12 | 92.15 | 94.23 | 98.14 | 94.15 | 98.12 |

NPV, negative predictive value; PPV, positive predictive value; SEN, sensitivity; SPE, specificity.
